# Predictive vision-language monitoring for proactive safety in robot task execution

**DOI:** 10.3389/frobt.2026.1870024

**Published:** 2026-08-03

**Authors:** Juan Diego Peña-Narvaez, Rodrigo Pérez-Rodríguez, Esther Aguado, Francisco Miguel Moreno, Francisco Martín Rico

**Affiliations:** 1 Intelligent Robotics Lab, Signal Theory, Communications, Telematics Systems, and Computation Department, International Doctoral School, Rey Juan Carlos University, Fuenlabrada, Spain; 2 Intelligent Robotics Lab, Universidad Rey Juan Carlos, Fuenlabrada, Spain

**Keywords:** AI-enabled robotics, human-robot collaboration, predictive monitoring, robot safety, task planning, vision-language models

## Abstract

Robots that execute language-conditioned tasks in dynamic environments often rely on feedback only after an action has failed, which can be insufficient when failures involve collisions or workspace conflicts. This paper presents a predictive monitoring framework that uses Vision-Language Models (VLMs) to assess near-future execution risk during robot task execution. The framework first generates structured plans with action execution conditions and a plan-level fallback action. During execution, a monitoring module combines visual observations, the current action, and the relevant execution conditions to estimate whether a condition is likely to be violated within a short future time window. When the predicted risk exceeds a task-specific threshold, the robot halts the current action, executes the fallback behavior, and replans from the updated state. We evaluate the approach in Gazebo simulation on mobile navigation with a moving human obstacle and manipulation with two robot arms sharing a workspace. Across controlled collision-risk settings, the proposed method achieves higher task success rates than reactive VLM-based baselines while requiring fewer replanning events than conservative current-state precondition checking. The results indicate that predictive vision-language monitoring can improve task completion in simulated dynamic robot tasks, while remaining subject to limitations such as VLM latency, prompt sensitivity, and evaluation beyond simulation.

## 1 Introduction

Recent advances in Large Language Models (LLMs) and Vision Language Models (VLMs) have enabled robots to interpret natural language instructions and decompose them into executable actions through semantic reasoning (Wei et al., 2022; Wang and Zhou, 2024; Ahn et al., 2022; Zhao et al., 2023; Jansen, 2020). However, current methods still face limitations in safety-oriented execution. Vision Language Action (VLA) models (Zitkovich et al., 2023; Kim et al., 2025) prioritize task success over safety objectives, lacking explicit constraints to reduce dangerous states (Zhang et al., 2025). Meanwhile, LLM-based planners, though capable of semantic reasoning, remain predominantly reactive (Huang et al., 2022; Yoneda et al., 2023; Mei et al., 2024; Guo et al., 2024; Kim et al., 2024), responding only after failures occur. In physical robotics, this “fail-then-recover” paradigm is unsuitable when the consequences are irreversible, such as robot-human collisions or hardware damage.

We introduce a predictive monitoring framework that shifts from reactive correction to proactive failure prevention. Our approach leverages VLMs to generate structured plans with embedded execution conditions and a plan-level fallback action, then continuously forecasts condition violations within a future time window during execution. When potential failures are predicted with sufficient confidence, the system immediately halts execution and activates the fallback action before physical errors occur.

**FIGURE 1:**
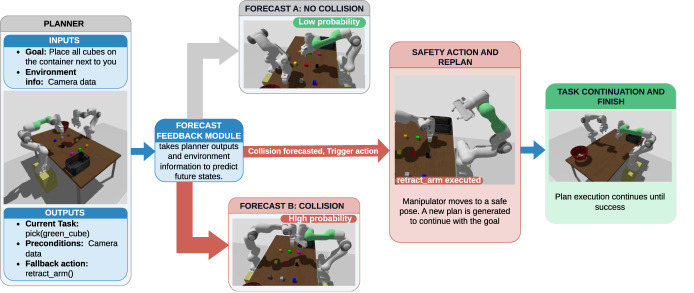
Overview of the predictive monitoring framework. The Planner generates a structured plan with embedded execution conditions and one plan-level fallback action from the task goal and environment state. During execution, the Forecast Feedback Module continuously monitors the current state and predicts potential condition violations within a future time window. When a collision is forecasted (Forecast B), the system proactively triggers the fallback action and initiates replanning, reducing the likelihood of physical failure. When no collision is predicted (Forecast A), execution continues until task completion.

The framework operates through two integrated components. A VLM-based planner generates an action sequence, predicts which execution conditions are at risk for the first executable action, and selects one fallback action for the plan. During execution, a predictive monitoring system continuously evaluates sensory inputs against the current action, using the VLM to forecast whether the relevant conditions will be violated within a specified time window (e.g., the next second). Risk assessment is formalized as an ordinal classification problem, where visual context and motion cues are mapped to semantic likelihood categories. When a predicted failure exceeds the confidence threshold, the system preemptively triggers the plan-level fallback and replans from the current state, supporting safer execution in dynamic scenes. Figure 1 shows an overview of our framework.

Experiments across navigation and manipulation tasks evaluate this approach, showing that predictive monitoring anticipates failures in dynamic scenarios and achieves higher task success rates than reactive baselines.

Our contributions are summarized as follows:

- A structured planning approach that generates action sequences with embedded execution conditions and a plan-level fallback action for VLM-based reasoning about potential execution risks.
- A predictive monitoring system that continuously forecasts execution-condition violations within a future time window using structured confidence levels, triggering the fallback action before physical failures occur rather than reacting after errors manifest.
- An experimental evaluation in navigation and manipulation tasks addressing two research questions: (1) Does predictive monitoring enable proactive failure prevention before physical errors occur? (2) Does our approach lead to higher task success rates compared to reactive baselines in dynamic environments with high collision probability?

## 2 Related work

Early work sought to bridge LLMs’ abstract knowledge with real-world constraints. SayCan (Ahn et al., 2022) pioneered this direction by combining LLM semantics with learned affordances for grounded plan selection. For generating executable instructions, ProgPrompt (Singh et al., 2023) introduced code-like templates with assertions, while FGTP (Li et al., 2024) enforced constraints using object ontologies. Further linking symbolic and semantic planning, LLM + P (Liu B. et al., 2023) used LLMs to define PDDL problems for classical planners. Although strong in semantic reasoning, these methods largely focus on initial plan generation rather than on adapting plans during execution.

To address dynamic environments, later work closed the perception-action loop. Inner Monologue (Huang et al., 2022) used textual feedback for replanning, and Statler (Yoneda et al., 2023) maintained a global state for long-term consistency. CoPAL (Joublin et al., 2024) extended this with multi-layer refinement to correct geometric or heuristic errors.

Semantic robot execution frameworks provide a complementary view by representing actions as skills whose applicability and continued validity are described through symbolic or semantic conditions. SkiROS2 (Mayr et al., 2023), for example, organizes ROS-based robot behaviors as skills with pre-, hold-, and post-conditions, supported by a knowledge base and behavior-tree-style execution. CRESTA (Gasperini et al., 2025) similarly emphasizes semantic-driven task awareness for monitoring and interpreting robot execution state. These frameworks motivate explicit execution-condition representations, but they do not address VLM-based visual forecasting of near-future condition violations.

A subsequent wave emphasized predictive and feedback-driven reactivity. Text2Reaction (Yang et al., 2024) introduced a structured “three-hop” reasoning cycle for error correction, while ISR-LLM (Zhou et al., 2024) employed iterative validators for feasibility. Visual grounding became central in approaches like LLM-Planner (Song et al., 2023) and PromptBook (Arenas et al., 2024), which used structured visual feedback for dynamic grounding. VILA (Hu et al., 2023) leveraged GPT-4 V to generate actionable steps directly from visual observations. For fine-grained error detection, ReplanVLM (Mei et al., 2024) and DoReMi (Guo et al., 2024) used VLMs as high-frequency constraint checkers, enabling systems like REFLECT (Liu Z. et al., 2023) to analyze sensory histories and explain failures.

Most closely related to proactive failure monitoring, Code-as-Monitor (Zhou et al., 2025) uses VLM-generated code to evaluate spatio-temporal constraints for reactive and proactive failure detection. Our work shares the goal of preventing foreseeable failures, but differs in its representation and control loop: rather than generating executable monitor code over explicit geometric constraints, the proposed framework prompts a VLM to assign ordinal risk categories to semantic execution conditions and couples this monitor to a plan-level fallback and replanning mechanism.

Predictive capabilities are also explored in world-model-based methods. VLWM (Chen et al., 2025) formalizes a System-2 loop to simulate future states and minimize semantic cost, while VLM-PC (Chen et al., 2024) forecasts outcomes for adaptive legged navigation.

Parallel advances in Vision-Language-Action (VLA) models, such as RT-2 (Zitkovich et al., 2023) and OpenVLA (Kim et al., 2025), unify perception and control in end-to-end architectures. Although effective, VLAs often operate as black boxes, making it difficult to inject explicit safety constraints or fallback behaviors, which is problematic for safety-critical robotics.

Our approach shifts from reactive to predictive behavior. Whereas detection-based methods (e.g., DoReMi, Text2Reaction) respond after failures, we generate failure-prone execution conditions and a plan-level fallback during initial plan creation. The predictive monitoring system forecasts failures within a future time window, halting execution and activating the fallback before errors occur.

## 3 Methods

### 3.1 Proposed framework

Our framework integrates two components: a VLM-based planner that generates action sequences with embedded execution conditions and one plan-level fallback action, and a predictive monitoring system that forecasts failures during execution. Given an instruction (e.g., *‘Find the bed’*), the planner generates future execution conditions for the first executable action and selects a fallback action for the plan. During execution, the monitoring system uses sensory inputs and the VLM to forecast condition violations within a future time window. When predicted failure exceeds the risk threshold, the system halts execution, activates the fallback, and replans from the current state until task completion. Algorithm 1 summarizes this flow.

Algorithm 1 Predictive Monitoring Framework

1: **Input:** Instruction I , initial state $S_{0}$ , risk threshold θ∈P , prediction horizon T

2: **Output:** Successful Task Completion
3: **Notation:** A : action sequence; $C_{0}$ : first-action execution conditions; f : fallback; p : risk category; θ : trigger threshold; T : prediction horizon
4: Generate initial plan $\Pi =\left\langle A,C_{0},f\right\rangle$ using VLM
5: Set current state $S\leftarrow S_{0}$ , current action index i←1

6: **for** each action $a_{i}\in A$ **do**
7:  Execute action $a_{i}$

8:  **while** action $a_{i}$ is not complete **do**
9:   Observe current state $S^{\prime }$ (image + robot state)
10:  Query VLM with $\left(S^{\prime },a_{i},C_{0},T\right)$ to obtain risk category p∈P

11:   **if** δ(p,θ)=1 **then**
12:    {Risk ≥ threshold}
13:    Halt execution of $a_{i}$

14:   Execute fallback action f

15:   Generate new plan $\Pi^{\prime }\leftarrow \left\langle A^{\prime },C_{0}^{\prime },f^{\prime }\right\rangle$ from state $S^{\prime }$

16:    Set i←1

17:    **break**
18:   **end if**
19:  **end while**
20:  Update current state $S\leftarrow S^{\prime }$

21:  Increment i

22: **end for**
23: **Return** Successful Task Completion

The algorithm operates as follows: Given a task instruction I and initial state $S_{0}$ , the VLM generates a plan Π (Equation 1) consisting of an action sequence $A=\left[a_{1},a_{2},\ldots ,a_{n}\right]$ , execution conditions $C_{0}$ for the first executable action, and a plan-level fallback action f that applies to the entire execution. We use the term execution conditions to include both preconditions that should hold before starting the action and holding conditions that should remain valid while the action is executing. During execution, the system continuously queries the VLM with the observed current state $S^{\prime }$ , current action $a_{i}$ , $C_{0}$ , and prediction time window T to obtain a risk category p∈P (Equation 2) (e.g., “very likely”, “likely”, “neutral”). The threshold function δ(p,θ) (Equation 4) evaluates whether the predicted risk exceeds the configured threshold θ . When triggered, the system halts execution, performs the fallback f , and generates a new plan from the current state. This cycle repeats until task completion.

Action completion is determined by the underlying ROS action interface used by each primitive. Navigation actions are executed through Nav2, which reports whether the navigation task succeeded, failed, or was canceled. Manipulation actions are executed through MoveIt/action execution, where trajectory execution status is mapped to the same task-result categories. The planner treats SUCCEEDED actions as completed and replans or advances from the updated state; FAILED actions trigger replanning with failure feedback; and CANCELED actions indicate that the forecast monitor requested an early stop, after which the plan-level fallback is executed before replanning. These action-status signals are not intended to provide a general semantic postcondition verifier. In the reported experiments, task-level failures are additionally recorded through the experiment monitors, including laser-based collision checks for navigation and arm-collision checks for manipulation. The manipulation simulation also uses Gazebo object poses to track whether cubes remain on the table or have reached a recipient, but the framework does not yet implement arbitrary semantic postconditions, such as detecting post-grasp slippage after a successful trajectory, unless such checks are explicitly encoded as task-specific conditions.

### 3.2 Plan generation

**FIGURE 2:**
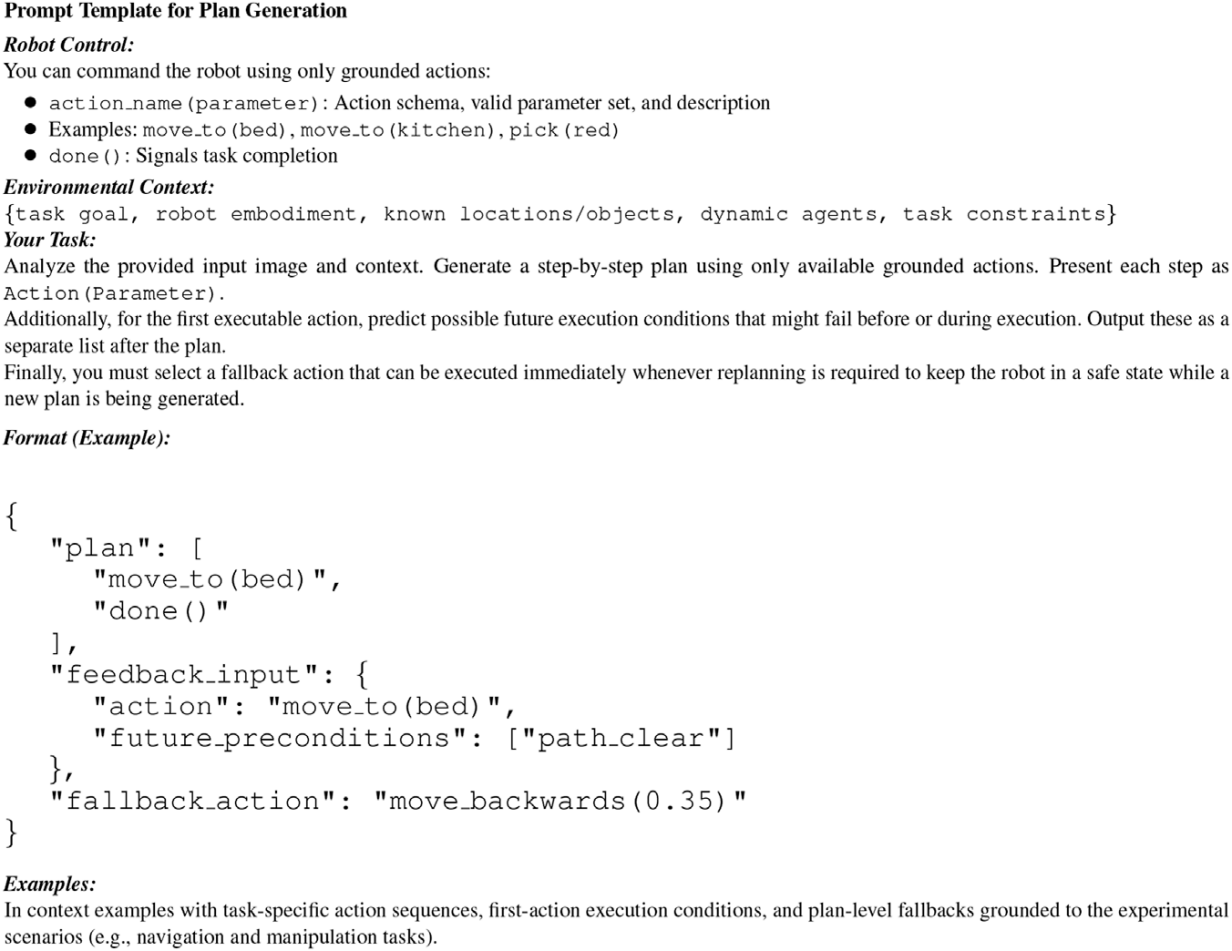
Overview of the prompt template used for the VLM.

For plan generation, we use a VLM to create structured action sequences from high-level instructions. Unlike approaches that only generate action sequences, our planner also specifies future execution conditions for the first executable action and selects one fallback action for the plan. For example, in navigation tasks, the planner generates conditions such as “path will remain clear” and a fallback action such as ”wait” or ”move_back” to be executed if violations are predicted. The prompt includes the task goal, current environment state (image), available actions with their API, environmental context, and in-context examples. Figure 2 shows the general structure of our plan generation prompt.

Action parameters are grounded by the environment context rather than generated as arbitrary free text. In navigation, valid parameters are known place symbols such as bed, kitchen, or sofa; in manipulation, valid parameters are visible or known object symbols such as cube colors. The environmental context comprises the robot embodiment, task goal, known objects and locations, relevant dynamic agents, and task constraints. For example, the navigation context specifies a Tiago robot in an apartment-like environment, reachable furniture locations, and a moving human that may cross the path; the manipulation context specifies the controlled green-marked robot arm, the independently moving second arm, visible cube colors, and workspace-interference constraints.

The VLM output is structured as JSON and parsed into the following data structure. Formally, a plan Π is represented as:

$$\Pi =\left\langle A,C_{0},f\right\rangle$$ (1)

where $A=\left[a_{1},a_{2},\ldots ,a_{n}\right]$ is the ordered sequence of actions, $C_{0}$ represents the execution conditions for the first executable action $a_{1}$ , and f is the single plan-level fallback action. Each action $a_{i}$ is encoded as a string in the form action_name(parameters). The conditions $C_{0}=\left\{c_{1},c_{2},\ldots ,c_{k}\right\}$ are semantic constraints predicted to be at risk during execution of $a_{1}$ . This representation enables the monitoring system to track which conditions to evaluate and which plan-level fallback to trigger if prediction indicates failure. The implementation-facing prompt fields retain the names future_preconditions and preconditions for parser compatibility; conceptually, these fields correspond to the execution-condition set $C_{0}$ used throughout this paper.

The Plan Generator is also used to replan from the current state whenever a predicted failure is detected by the predictive monitoring system. In this case, the prompt is augmented with the current sensory input and the selected fallback action as the new starting point for generating an updated plan. This conditions the replanned actions on the current environment rather than only on the initial state.

### 3.3 Predictive monitoring

The predictive monitoring system forecasts potential failures in the execution of the generated plan before they occur. This proactive mechanism allows the robot to adapt its behavior during task execution.

To achieve this, we leverage a VLM that can interpret the robot’s sensory inputs (e.g., camera images) in conjunction with the planned actions and their associated execution conditions. The VLM is prompted to evaluate whether any predicted condition is likely to be violated within a specified future time window, based on the current state of the environment.

The monitoring process operates during plan execution. At each monitoring step, the system captures the current sensory input and combines it with the current action in the plan and its execution conditions. This information is fed into the VLM, which analyzes the data to predict potential failures. Rather than forcing binary predictions (fail/succeed), we elicit ordinal risk assessments from the VLM through structured prompts that guide it to output semantic categories of failure likelihood (e.g., “very likely,” “likely,” “neutral,” “improbable,” “very improbable”) based on environmental observations and predicted motion dynamics. This categorical output provides sufficient granularity for threshold-based decision-making.

**FIGURE 3:**
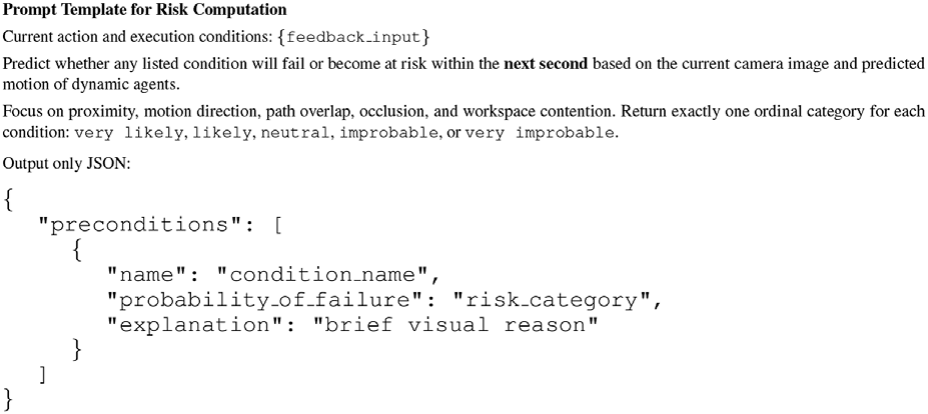
Overview of the prompt template used for VLM risk computation.

Concretely, each monitoring query consists of a single camera frame paired with a textual prompt that specifies the current action, the at-risk execution conditions, and the prediction time window. The system does not pass frame histories, optical flow, or explicit velocity estimates to the VLM. Instead, it relies on the VLM’s pretrained capacity to infer motion dynamics from visual cues observable in individual frames, including agent pose, limb orientation, relative proximity, and spatial arrangement. The prompt instructs the VLM to reason about whether dynamic agents in the scene are likely to interfere with the current action within the next second, effectively delegating temporal extrapolation to the model’s internal visual reasoning (Liu et al., 2025). While this single-frame design limits the temporal information available to the model, it simplifies the monitoring pipeline and avoids the latency overhead of multi-frame buffering. Figure 3 summarizes the prompt template used for risk computation.

The ordinal categories allow the system to distinguish between different degrees of risk, supporting task-specific threshold configuration. For instance, safety-critical navigation tasks can use a conservative threshold (“neutral”), while manipulation tasks with recoverable errors can tolerate higher risk thresholds (“likely”). Importantly, we do not claim these categories represent calibrated probability estimates and acknowledge that VLMs can produce overconfident or inconsistent risk assessments. The semantic labels function as structured heuristics rather than precise failure probabilities. The threshold operator in Equation 4 treats the categories as an ordered scale and does not require specific numeric probability values; what matters for correct operation is that the VLM maintains a monotonic relationship between its risk output and actual collision likelihood, not that each category maps to a fixed probability range. In our experiments, the discrimination between risk levels proved sufficient for threshold-based decisions, as supported by the ablation results in Table 4, where removing prediction capability caused the largest performance degradation.

**TABLE 4:** Ablation study results for navigation task at p=0.8 . Results show mean ( ± std) over 100 trials.

Variant	Success rate (%) ↑	Replan count ↓
Full system	**89(** ± **31)**	**7.5(** ± **6.0)**
W/o prediction	18 ( ± 39)	0.1 ( ± 0.5)
W/o fallbacks	80 ( ± 40)	11.2 ( ± 8.3)
W/o preconditions	70 ( ± 46)	25.3 ( ± 24.5)
Time window = 0.5s	76 ( ± 43)	8.1 ( ± 6.5)
Time window = 2.0s	89 ( ± 30)	7.6 ( ± 6.1)
Time window = 3.0s	89 ( ± 31)	7.5 ( ± 6.0)

Bold values indicate the best result.

At each step of action execution, the predictive monitoring system evaluates the current environment and motion dynamics, asking the VLM to assess whether any execution condition for the current action is at risk. Specifically, the prompt includes the current state and the task’s conditions, with the goal of forecasting the likelihood category for condition violation within a given time window.

**FIGURE 4:**
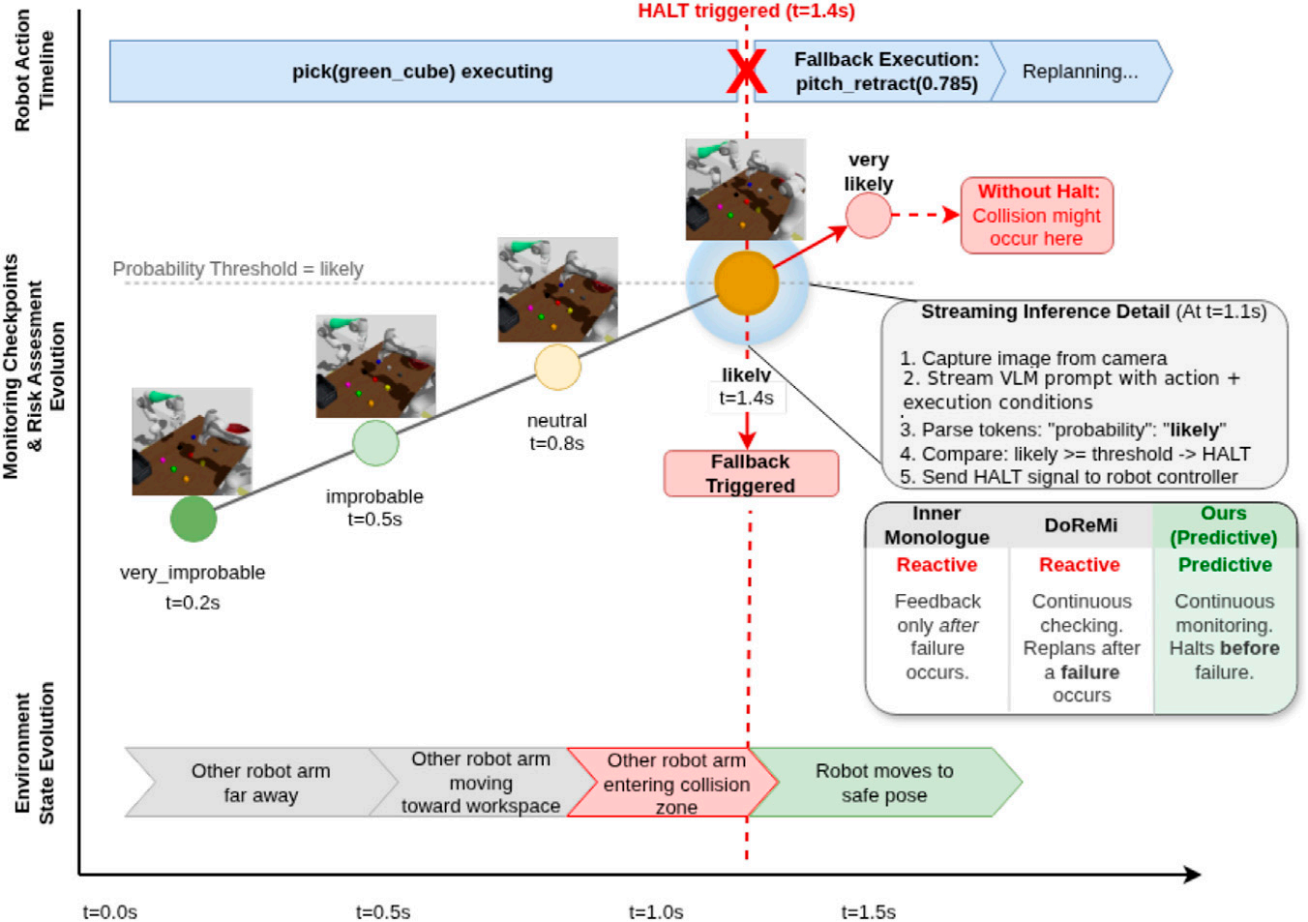
Predictive monitoring workflow showing temporal evolution of risk assessment during a manipulation task. As environmental risk increases (another robot arm approaching), the system forecasts a collision and triggers fallback at t=1.4 s before physical contact. Bottom panel contrasts predictive vs. reactive baseline behaviors.

The prompt in Figure 3 is designed to focus on key factors that influence condition validity, such as proximity, motion direction, and workspace contention. Based on these inputs, the VLM predicts whether the condition will hold or fail, outputting a risk category that indicates the likelihood of failure. Figure 4 illustrates this workflow: as another robot arm approaches the workspace, the predicted risk category transitions from “very improbable” ( t=0.2 s) to “likely” ( t=1.4 s), triggering immediate halt and fallback execution before collision occurs. This contrasts with reactive baselines that only respond after failures manifest.

To enable systematic decision-making during monitoring, we formalize the risk categories and thresholding mechanism. The VLM outputs a discrete risk category p∈P , where:

$$P=\left\{\text{very improbable},\text{improbable},\text{neutral},\text{likely},\text{very likely}\right\}$$ (2)

with an ordering relation defined as:

very improbable<improbable<neutral<likely<very likely (3)

For implementation, we map each category to an integer value following the ordering in Equation 3: very improbable=1 , improbable=2 , neutral=3 , likely=4 , and very likely=5 , enabling efficient comparison operations.

The fallback trigger decision is formalized through a threshold operator. Given a configured threshold θ∈P , the system triggers a fallback when:

$$\delta \left(p,\theta \right)=\left\{\begin{array}{cc} 1 & \text{if }p\geq \theta \\ 0 & \text{otherwise} \end{array}\right.$$ (4)

where δ(p,θ)=1 causes immediate action halt and fallback activation. In our experiments, we use θ=likely for manipulation tasks and θ=neutral for navigation tasks. The choice of threshold reflects a trade-off between intervention sensitivity and unnecessary replanning. A lower threshold such as “neutral” causes the system to intervene at earlier signs of risk, which is appropriate for navigation where collisions with humans are non-recoverable and reaction margins must account for the robot’s stopping distance and VLM inference latency. A higher threshold such as “likely” is suitable for manipulation where workspace contention between robot arms is frequent but often transient, and overly conservative intervention would trigger excessive replanning without proportional safety benefit. In representative manipulation logs, neutral-risk predictions occurred frequently during benign shared-workspace proximity; replaying the trigger rule on 668 logged ordinal outputs would produce 197 interventions at θ=neutral but only one intervention at θ=likely . This supports the use of a less conservative threshold in manipulation, where transient proximity is common. The selected thresholds should therefore be interpreted as task-specific operating points rather than globally optimal values. A closed-loop threshold sensitivity study confirming this choice for the manipulation domain is presented in Section 4.5.2.

The VLM uses streaming inference to generate the JSON response incrementally. As tokens are streamed, the system parses the risk category from the JSON field ”probability_of_failure”. When the parsed category p satisfies δ(p,θ)=1 , the system immediately halts action execution and activates the fallback strategy without waiting for the complete response.

In continuous monitoring mode, the planner starts the feedback monitor before executing the current action. The planner execution cycle is driven by a 1.0 s timer, while the feedback node checks whether an active image and prompt are available every 0.25 s. The effective decision rate is bounded by VLM inference latency; in Section 4.4.1, the first parsed risk category is available after 1.14 ( ± 0.28) s on representative manipulation frames. In the reported experiments, each active action used one at-risk execution condition, such as path_clear for navigation or trajectory_clear for manipulation. Therefore, conditions were not queried sequentially and no mean-risk aggregation was required; each monitoring call produced one ordinal risk decision for the current action.

## 4 Experiments

### 4.1 Environment and setup

**FIGURE 5:**
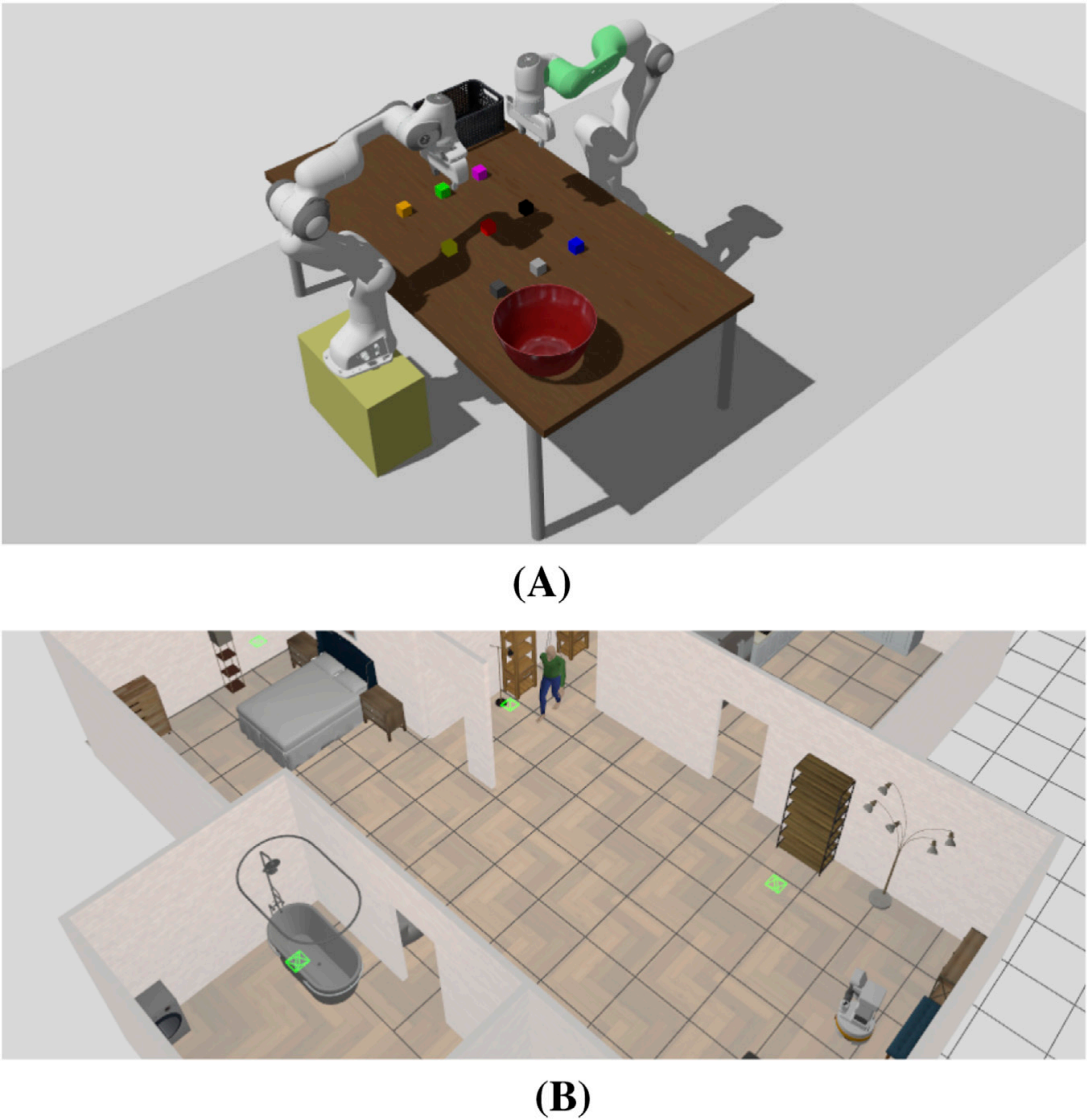
Experimental environments for manipulation **(A)** and navigation **(B)** tasks.

We evaluate our predictive monitoring framework in both navigation and manipulation tasks. Since our approach focuses on predictive methods, we specifically test the framework in dynamic environments. All experiments are conducted in the Gazebo simulation environment. For navigation, we use a PAL Robotics Tiago mobile robot in a scenario where a human moves around the workspace, creating dynamic obstacles that require proactive collision avoidance. For manipulation, we employ a Franka Emika Panda robotic arm in a cooperative task where a second Panda arm operates independently and does not share its state or intentions with our agent, resulting in unpredictable workspace changes and potential interference. The illustrated environments for both tasks are shown in Figure 5.

To systematically evaluate our framework under dynamic conditions, we introduce controlled collision probabilities $p_{\mathrm{collision}}\in \left\{0.2,0.5,0.8\right\}$ in both environments.

For the navigation task, the robot finds and reaches furniture in a simulated house while a human agent walks along a predefined trajectory. At trial start, the human spawns at a collision pose (intersecting the robot’s path) with probability $p_{\mathrm{collision}}$ , otherwise at a safe pose. Collision detection uses the laser scanner (readings within ±45° forward, range [0.1 m, 0.5 m]), terminating trials as failures.

For the manipulation task, our controlled agent (Robot1) places colored cubes into a recipient while a second arm (Robot2) independently executes its own pick-and-place routine without communication. With probability $p_{\mathrm{collision}}$ , Robot2 targets the same first cube as Robot1, creating workspace contention. Otherwise, Robot2 attempts simple coordination through imperfect trajectory observation.

We conducted approximately 100 independent trials per method and probability setting. Visual inputs were task-specific: head-mounted camera (first-person view) for navigation, fixed overhead camera for manipulation. We implemented our framework using gemini-2.5-flash for planning and gemini-2.5- flash-lite for monitoring, prioritizing reasoning capability for planning and response latency for monitoring.

We additionally conducted a targeted navigation stress test with three moving people in the same simulated house. This stress test evaluates the full predictive monitoring system under the hardest navigation setting $\left(p_{\mathrm{collision}}=1.0\right)$ , where every trial is initialized with pedestrian motion that can intersect the robot path. Because this experiment was designed to test scalability of the proposed monitor rather than to re-run the full baseline suite, it is reported separately from the main comparison.

The code repository is available^1^, including video demonstrations of the predictive monitoring system in both navigation and manipulation scenarios. The feedback prompt template, representative ROS logs, and timestamp-matched manipulation frames are provided in Supplementary Material S1.

### 4.2 Baselines

To evaluate the effectiveness of our predictive monitoring framework, we compare it against two baselines that represent complementary paradigms in LLM/VLM-based robotic execution. We select Inner Monologue (Huang et al., 2022) as a foundational reactive approach that pioneered closed-loop feedback for LLM planning, and DoReMi (Guo et al., 2024) as the most similar method to ours that uses VLM-based precondition monitoring. All methods share the same action execution pipeline, sensory inputs, and models; however, they differ in their strategies for detecting and responding to errors during task execution.

- Inner Monologue (IM) (Huang et al., 2022): This reactive approach employs a VLM as both a scene descriptor and a success detector to provide feedback after each action execution. For planning, the VLM receives an image and generates the next single action using an “inner monologue” strategy that analyzes feedback from previous action outcomes. For monitoring, a separate VLM evaluates whether the executed action succeeded by analyzing the current scene (e.g., “Is the cube in the gripper?” for pick(color)). The system outputs binary Yes/No success signals. Upon detecting failure, the planner generates a new action based on the failure description. This method only responds *after* a failure has already occurred, making it unable to prevent physical errors from manifesting.
- DoReMi (Guo et al., 2024): This method uses a VLM to detect plan execution misalignment by checking whether action preconditions are satisfied in the *current* state. The planner generates a full action sequence with preconditions for the first action (similar to our approach but without a plan-level fallback). During execution, the monitoring VLM receives the current image and preconditions, then evaluates: “Is this precondition currently violated?” (e.g., “Is the path currently blocked?”). When a violation is detected in the present state, the system triggers replanning. While DoReMi is the closest baseline to our framework, it remains fundamentally reactive: it evaluates preconditions at the present moment rather than forecasting their validity in the near future. Consequently, it can only respond to failures that have already begun to manifest, rather than preventing them proactively.

In contrast, our approach forecasts execution-condition violations within a future time window by prompting the VLM to predict agent motion and assess risk categories (very likely, likely, etc.), enabling the system to halt execution and activate the plan-level fallback *before* physical failures occur. This distinction between checking the current state versus predicting future states represents the core contribution of our work.

### 4.3 Evaluation metrics

We evaluate the predictive monitoring framework using three metrics. Success Rate (%) measures the percentage of trials in which the robot successfully completes the assigned task–reaching the target furniture without colliding with the moving human for navigation, or placing all cubes into the recipient without workspace conflicts for manipulation. Execution Time (s) captures the total time from task initiation to completion, including overhead introduced by monitoring and replanning; lower times indicate more efficient execution. Replan Count measures the average number of replanning events triggered during successful task executions (Zhao et al., 2025), revealing the frequency of adaptive behavior; lower counts suggest fewer unnecessary replans, while zero replans may indicate a lack of adaptivity. Both Execution Time and Replan Count are measured exclusively on successful trials. Since our experimental environments are designed such that unsuccessful trials represent non-recoverable errors (e.g., collision with a human, workspace conflicts), failures can occur at any point during execution, from the beginning with zero replans to late in the task with many replans, resulting in high variability. Therefore, we report these metrics only for successful trials to ensure meaningful comparisons. For success-rate comparisons, we additionally computed Wilson 95% confidence intervals and two-proportion z-tests between our method and each baseline.

For each experimental condition (task type and collision probability), we report the mean and standard deviation over independent trials.

### 4.4 Results and discussion

**FIGURE 6:**
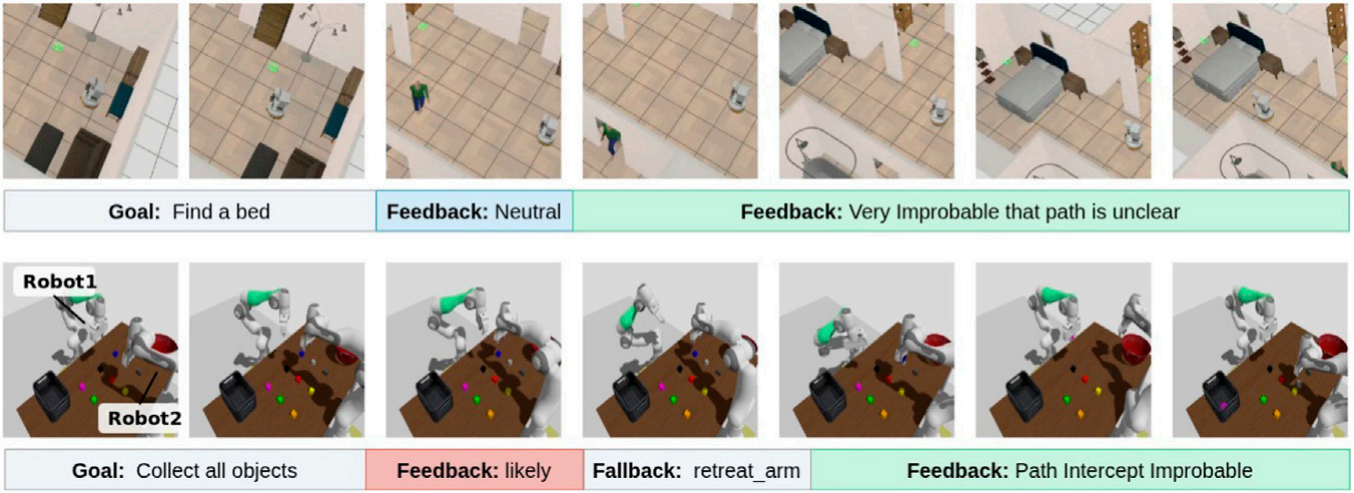
Execution trace of the predictive monitoring system showing action timeline, predicted-risk flags, and fallback activations during a single trial. In the bottom row, Robot1 denotes the green-marked controlled manipulator and Robot2 denotes the independently moving manipulator.

Figure 6 illustrates the execution trace from representative trials in both tasks. The top row shows a navigation scenario where the robot’s goal is to find a bed. As the robot navigates, the predictive monitoring system continuously evaluates risk: initially outputting “Neutral” and “Very improbable that path is unclear” when the environment is safe, indicating low collision risk with the moving human. The bottom row demonstrates a manipulation task where Robot1 must collect all objects while Robot2 operates independently. As Robot2’s arm approaches the shared workspace (visible in the third frame), the monitoring system forecasts increasing collision risk, transitioning from safe predictions to “Feedback: likely” (highlighted in red), which exceeds the configured threshold. This triggers immediate execution halt and activates the fallback action ”retreat_arm” before physical contact occurs. After the fallback, replanning generates a new plan, and monitoring resumes with a low-risk path-intercept prediction, confirming successful collision avoidance. This sequence demonstrates the system’s ability to forecast and prevent failures proactively, contrasting with reactive methods that would only respond after collision.

Table 1 presents the success rate comparison across all experimental conditions. Monologue shows moderate success rates only in low collision probability scenarios ( p=0.2 : 80% navigation, 84% manipulation), but these successes occur primarily in trials where the dynamic obstacle never enters the robot’s path, allowing execution to complete without interference. In our experimental setup, failures are irrecoverable (e.g., collision with a human in navigation, workspace interference in manipulation), causing immediate trial termination. Since Monologue operates reactively, it can only respond after a failure has already occurred, which is too late to avoid trial termination. As collision probability increases, Monologue’s success rate drops sharply (17%–21% at p=0.8 ) because it does not anticipate these failures before they manifest physically. DoReMi demonstrates moderate performance (76%–81% for navigation, 65%–80% for manipulation), maintaining relatively stable success rates even at high collision probabilities through conservative behavior that triggers replanning whenever preconditions are violated in the current state. While this conservatism helps avoid some failures, it does not distinguish between imminent threats and distant obstacles that may not actually lead to collisions. In contrast, our method achieves the highest success rates across all conditions, indicating that predictive monitoring anticipates failures and improves execution outcomes in simulation. In particular, as the probability of collision increases, the performance gap between our approach and the baselines widens. For navigation at p=0.8 , while Monologue drops to 17% success, our method maintains 89%, representing a 5.2 × improvement. This supports our first research question: predictive monitoring reduces observed collision-related failures in the evaluated simulation scenarios.

**TABLE 1:** Success rate comparison under different collision probabilities. Results show percentage ( ± binomial std) over independent trials.

Task	$p_{\mathrm{collision}}$	Monologue	Ours	DoReMi
Navigation	p = 0.2	80 ( ± 40)	**95(** ± **22)**	81 ( ± 39)
Navigation	p = 0.5	42 ( ± 50)	**90(** ± **30)**	77 ( ± 42)
Navigation	p = 0.8	17 ( ± 38)	**89(** ± **31)**	76 ( ± 43)
Manipulation	p = 0.2	84 ( ± 37)	**93(** ± **26)**	80 ( ± 40)
Manipulation	p = 0.5	54 ( ± 50)	**80(** ± **40)**	71 ( ± 46)
Manipulation	p = 0.8	21 ( ± 41)	**72(** ± **45)**	65 ( ± 48)

Bold values indicate the best result.

Statistical tests support the navigation improvements: our method outperformed both Monologue and DoReMi at all navigation risk levels ( p<0.016 in all pairwise comparisons). For manipulation, the improvement over Monologue was significant at p=0.5 and p=0.8 $\left(p<10^{-4}\right)$ , and the improvement over DoReMi was significant at p=0.2 (p=0.008) . The advantage over DoReMi at higher manipulation risk remained positive ( p=0.139 at p=0.5 , p=0.287 at p=0.8 ), indicating that predictive monitoring provides the clearest gains in navigation and remains competitive in dual-arm manipulation.

Table 2 shows execution time and replan count metrics. Monologue achieves the lowest execution times among successful trials, which reflects that these cases involved no collisions and no replanning. However, with very low success rates at high collision probabilities (17%–21%), these represent only a small fraction of trials where environmental conditions happened to be favorable. DoReMi exhibits the highest and most variable execution times due to its conservative replanning strategy, which triggers even when violations may not lead to actual failures. In comparison, our method achieves competitive execution times (second lowest) while maintaining higher success rates across all conditions, suggesting that predictive monitoring avoids unnecessary replanning by differentiating between high-risk and low-risk condition violations.

**TABLE 2:** Xecution time and replan count comparison. Results show mean ( ± std) computed over successful trials only.

Task $\left(p_{\mathrm{collision}}\right)$	Monologue	Ours	DoReMi
Execution time (s)			
Nav (0.2)	52.7 ( ± 0.9)	61.5 ( ± 5.1)	76.9 ( ± 34.8)
Nav (0.5)	52.2 ( ± 3.3)	59.7 ( ± 15.6)	68.2 ( ± 37.2)
Nav (0.8)	52.2 ( ± 6.4)	56.8 ( ± 14.4)	71.0 ( ± 36.9)
Manip (0.2)	233.5 ( ± 62.6)	261.0 ( ± 33.9)	289.6 ( ± 71.1)
Manip (0.5)	225.3 ( ± 67.0)	237.7 ( ± 87.6)	262.5 ( ± 91.3)
Manip (0.8)	254.1 ( ± 41.5)	227.3 ( ± 97.6)	232.1 ( ± 94.6)
Replan count			
Nav (0.2)	0.0 ( ± 0.0)	7.7 ( ± 6.5)	23.6 ( ± 39.0)
Nav (0.5)	0.0 ( ± 0.0)	8.2 ( ± 6.7)	25.9 ( ± 25.1)
Nav (0.8)	0.0 ( ± 0.0)	7.8 ( ± 6.1)	25.6 ( ± 25.1)
Manip (0.2)	4.2 ( ± 2.4)	14.3 ( ± 9.5)	25.0 ( ± 14.7)
Manip (0.5)	4.6 ( ± 2.3)	11.4 ( ± 8.5)	21.4 ( ± 13.1)
Manip (0.8)	4.6 ( ± 2.2)	10.6 ( ± 9.3)	17.7 ( ± 14.6)

The Replan Count reveals differences in adaptivity. Monologue exhibits zero replans for navigation trials, reflecting a lack of adaptivity rather than optimal performance. DoReMi exhibits the highest replan counts (23.6–25.9 for navigation, 17.7–25.0 for manipulation) with substantial variance, consistent with conservative behavior that frequently triggers false-positive current-state violations. In contrast, our method shows moderate replanning frequencies (7.7–8.2 for navigation, 10.6–14.3 for manipulation), balancing adaptivity and stability. These replans are triggered before failures occur, allowing proactive fallback execution.

Comparing navigation and manipulation reveals task-specific characteristics. Manipulation tasks generally show higher success rates at low collision probabilities, but this advantage diminishes at p=0.8 , suggesting workspace contention is more challenging to handle reactively. Execution times are longer for manipulation (225–290s vs. 52–77s for navigation) due to pick-and-place complexity, but the relative performance ordering remains consistent across tasks.

#### 4.4.1 VLM feedback latency

Because our monitoring module relies on cloud VLM inference, we additionally measured the runtime latency of the streaming feedback path on 500 frames sampled from ten representative manipulation sequences. For each frame, we queried the monitoring prompt with the current action pick(black) and the at-risk execution condition trajectory_clear. We measured the time to the first streamed token, the time to the first parsed risk category, and the total response time. Table 3 summarizes these measurements.

**TABLE 3:** Streaming VLM feedback latency on representative manipulation frames. Results are in seconds and reported as mean ( ± std).

Metric	Samples	Latency (s)
First streamed token	500	1.01 ( ± 0.29)
First parsed risk category	500	1.14 ( ± 0.28)
Full response	500	4.37 ( ± 1.14)

These measurements show that streaming inference allows the system to extract the risk category well before the complete response is generated, reducing the effective monitoring latency from 4.37s for a full response to 1.14s for the first parsed risk category. This latency is still too slow for low-level servo control, indicating that the current cloud-based implementation is better suited to high-level task monitoring and fallback triggering. This motivates the local or hybrid VLM deployment strategies discussed in the limitations.

### 4.5 Ablation studies

To understand the contribution of individual components in our predictive monitoring framework, we conduct ablation studies on the navigation task with the highest collision probability (p=0.8) , where component differences are most pronounced. We evaluate the impact of prediction capability, the plan-level fallback action, structured condition monitoring, and prediction time windows. Table 4 presents the results, measuring Success Rate and Replan Count over 100 trials per variant. We report only these two metrics because execution time differences were marginal across ablations and would not provide additional insight within our page constraints.

We test the following variants: (1) *w/o Prediction* removes future forecasting, evaluating conditions only in the current state (similar to DoReMi), (2) *w/o Fallbacks* eliminates the plan-level safe action during replanning triggers, (3) *w/o Preconditions* removes the structured condition list supplied to the monitor, relying solely on general scene assessment, and (4) *Time Window variations* evaluate different prediction horizons (2.0s, 3.0s) against our default 1.0s window.

The ablation results show a clear performance hierarchy among components. *w/o Prediction* causes the largest degradation, dropping the success rate to 18% (comparable to reactive baselines like Inner Monologue), confirming that forecasting future failures rather than detecting current violations is the core enabler of proactive prevention. *w/o Fallbacks* reduces success to 80% with increased replanning (11.2 vs. 7.5 events), indicating that the immediate plan-level safe action provides a 9 percentage point improvement by maintaining robot safety while new plans are generated. *w/o Preconditions* results in 70% success with excessive replanning (25.3 events), demonstrating that structured condition reasoning prevents unnecessary conservative triggers by focusing monitoring on semantically relevant risks rather than general scene changes. Time window analysis shows that 0.5s horizons provide insufficient lead time for collision avoidance (76% success), while longer windows (2.0s, 3.0s) offer no additional benefit over our default 1.0s, indicating that 1.0s provides the best observed balance between early warning and prediction accuracy for the navigation dynamics tested here. Overall, these results show that predictive monitoring, the plan-level fallback action, and condition-based reasoning each contribute to performance in dynamic environments.

#### 4.5.1 Navigation-only geometric predictor diagnostic

To further separate general predictive capability from VLM-specific semantic reasoning, we ran an additional navigation-only diagnostic using a time-to-collision (TTC) heuristic. This diagnostic is not included in the main baseline comparison because it uses a different sensing modality and a simplified control protocol: the robot follows a fixed plan, move_to(bed) followed by done(), and the monitor uses only the forward laser scan sector. At each scan, the heuristic selects the closest valid range, estimates closing speed from consecutive scan updates, and computes $TTC=\left(d-d_{\mathrm{safe}}\right)/v_{\mathrm{closing}}$ when the obstacle is approaching, with $d_{\mathrm{safe}}=0.5$ m. If the estimated TTC falls below 1.0s, the current navigation action is cancelled and the robot executes back_up(0.5) before retrying the goal. Table 5 summarizes the diagnostic results for collision-risk and safe navigation trials.

**TABLE 5:** Navigation-only TTC diagnostic. Execution time and replan count are reported as mean ( ± std) over successful trials.

Condition	Valid trials	Success rate	Execution time (s)	Replan count
$p_{\mathrm{collision}}=1.0$	27	18/27 (66.7%)	68.1 ( ± 11.1)	2.5 ( ± 2.3)
$p_{\mathrm{collision}}=0.0$	30	30/30 (100.0%)	49.7 ( ± 0.4)	0.0 ( ± 0.0)

The TTC diagnostic shows that a simple geometric predictor can be useful for the local navigation collision case: it recovers 18 of 27 valid collision-risk trials and produces no unnecessary replans across 30 safe trials. At the same time, its scope is much narrower than the proposed VLM monitor. TTC only reasons about immediate range dynamics in the laser sector; it cannot evaluate semantic execution conditions, workspace contention, object or task state, partial observability, or non-geometric failures.

#### 4.5.2 Threshold sensitivity analysis for manipulation

To provide closed-loop evidence for the manipulation threshold choice, we ran additional experiments varying the trigger threshold θ∈{neutral,likely,very likely} under safe $\left(p_{\mathrm{collision}}=0.0\right)$ and collision-risk $\left(p_{\mathrm{collision}}=1.0\right)$ conditions. To isolate the effect of threshold selection from task-length variability, these experiments use a simplified two-cube configuration rather than the full nine-cube task used in the main evaluation. This reduction shortens each trial while preserving the same workspace-contention dynamics and monitoring pipeline. Table 6 summarizes the results.

**TABLE 6:** Threshold sensitivity for the manipulation task (two-cube configuration). Results show success rate (%) and mean replan count over successful trials.

Threshold (θ)	$p_{\mathrm{collision}}$	Success (%)	Replans
Neutral	0.0	80.0	7.2 ( ± 7.3)
Neutral	1.0	94.4	10.4 ( ± 8.5)
Likely ⋆	0.0	**100.0**	3.2 ( ± 3.8)
Likely ⋆	1.0	92.9	7.1 ( ± 6.4)
Very likely	0.0	66.7	0.7 ( ± 1.0)
Very likely	1.0	93.8	4.0 ( ± 4.9)

⋆ denotes the threshold used in the main experiments.

Bold values indicate the best result.

Collision-avoidance success is comparable across all three thresholds (92.9%–94.4%), indicating that the ordinal risk categories reliably discriminate genuine collision situations regardless of the trigger level. The critical differentiator is safe-trial stability. At θ=neutral , the system intervenes too frequently in the absence of real threats: nine of 12 successful safe trials included false-positive replans (mean 7.2 per trial), and three of 15 safe trials failed outright due to replanning-induced task-state confusion. At θ=very likely , intervention is too rare: only 0.7 replans per safe trial limits the system’s ability to recover from VLM planning errors, yielding only 66.7% safe success. The selected θ=likely achieves the best balance: it is the only threshold with 100% safe success while maintaining 92.9% collision-risk success, confirming its use as the manipulation operating point. These closed-loop results complement the offline replay analysis in Section 3.3 and confirm that threshold selection materially affects system behavior.

#### 4.5.3 Multi-person navigation stress test

**FIGURE 7:**
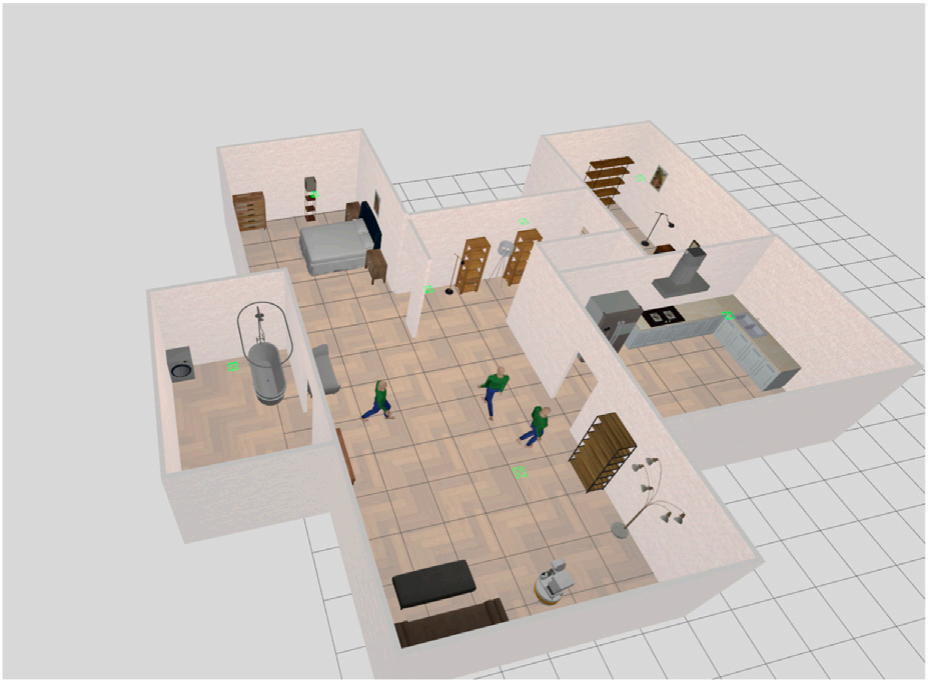
Three-person navigation stress-test scene. The Tiago robot navigates through the simulated house while three scripted moving people traverse paths that can intersect the robot’s route.

To probe the navigation setting beyond the single-person trials used in the main baseline comparison, we added two additional moving people to the simulated house, producing three independent dynamic agents in the robot workspace (Figure 7). All stress-test trials used $p_{\mathrm{collision}}=1.0$ , so each run started from a configuration in which pedestrian motion could obstruct the robot’s path to the goal.

Table 7 shows that the full system completed 148 of 200 stress-test trials (74.0%; Wilson 95% CI: 67.5%–79.6%). Performance is lower than in the main single-person navigation experiment at p=0.8 , as expected from the increased obstacle density and the all-collision initialization. Nevertheless, the system still resolves most trials by repeatedly forecasting path-intersection risk, activating the fallback, and replanning. This result provides additional evidence that predictive monitoring remains useful when the navigation scene contains multiple moving agents, while also confirming that dense pedestrian settings are more demanding for a cloud-VLM monitor with approximately 1 s decision latency.

**TABLE 7:** Multi-person navigation stress test with the full predictive monitoring system. Execution time and replan count are reported as mean ( ± std) over successful trials.

Scenario	Trials	$p_{\mathrm{collision}}$	Success rate	Execution time (s)	Replan count
Three moving people	200	1.0	148/200 (74.0%)	65.7 ( ± 13.2)	9.8 ( ± 3.4)

### 4.6 Limitations

While our results show improvements in success rates, several limitations merit consideration. Our reliance on cloud-based VLM APIs introduces latency, constraining applicability to high-frequency control tasks. Future work could explore local VLM deployment or hybrid architectures combining learned predictive models with VLMs for higher-level reasoning. Additionally, our simple low-level action primitives (move_to, pick, place) facilitate stable planning but limit extensibility to dexterous manipulation requiring fine-grained control, such as in-hand reorientation or contact-rich assembly. The current implementation also does not provide a general semantic postcondition checker for all possible post-action events, such as object slippage after a reported grasp trajectory. Our method exhibits conservative behavior, occasionally triggering the fallback when failures may not occur, reflected in moderate replan frequencies (7.7–8.2 for navigation, 10.6–14.3 for manipulation) and slightly increased execution times. However, this conservatism is useful for safety-oriented applications: while conservative predictions may slow execution in low-risk situations, they reduced observed collision-related failures in our simulations, maintaining 89% success for navigation and 72%–93% for manipulation even at high collision probabilities, compared to 17%–21% for reactive baselines.

All experiments were conducted in Gazebo simulation, where dynamic agents follow scripted or deterministic trajectories. Real-world environments introduce substantially greater motion variability: humans change direction abruptly, move at irregular speeds, and exhibit socially complex behaviors that a single-frame VLM observation may not fully anticipate. The main comparative navigation evaluation uses one moving person to establish a controlled baseline for proactive failure prevention, and the additional three-person stress test indicates that performance degrades as obstacle density increases. Handling dense environments with multiple moving pedestrians shifts the challenge toward high-frequency reactive collision avoidance, which is constrained by the current ∼ 1.14s reasoning latency of cloud-based VLMs. The 1-s prediction window, which proved effective in our simulated environments where motion dynamics are smooth and predictable, may require adjustment for settings with less structured agent behavior. The ablation results in Table 4 show that extending the window to two or 3 s yields no additional benefit in simulation, but this finding should not be assumed to generalize to scenarios with more erratic dynamics. Scaling the evaluation to physical robot platforms with real human participants remains an important step for establishing practical applicability.

## 5 Conclusion

We presented a predictive monitoring framework that shifts robotic task execution from reactive failure recovery toward proactive failure reduction. By leveraging VLMs to forecast execution-condition violations within a future time window, our approach enables robots to anticipate risky execution states before they occur. Our framework generates structured plans with a plan-level fallback action and continuously monitors execution through structured ordinal risk assessment, triggering safe responses when failures are predicted.

Experiments across navigation and manipulation tasks show improvements over reactive baselines. At high collision probabilities (p=0.8) , our method achieves 89% success in navigation compared to 17% for reactive approaches, and an additional three-person navigation stress test achieves 74% success under all-collision initialization. Ablation studies indicate that predictive capability is the primary contributor and that the plan-level fallback provides an immediate safety response during replanning. While API latency and simple action primitives currently limit applicability to high-frequency and more complex tasks, the framework provides a basis for safer robot operation in dynamic, uncertain environments where failure prevention is important.

## Data Availability

The datasets presented in this study can be found in online repositories. The names of the repository/repositories and accession number(s) can be found below: https://github.com/juandpenan/ros2_feedback_planner.

## Footnotes

1
https://github.com/juandpenan/ros2_feedback_planner
