# Supplementary material for: Predictive vision-language monitoring for proactive safety in robot task execution
Source: Front Robot AI. 2026 Aug 3;13:1870024. doi: 10.3389/frobt.2026.1870024 (PMC13477910; doi:10.3389/frobt.2026.1870024)
Supplement: Supplementary file 1 [file DataSheet1.pdf]

Supplementary Material

1 S1. OVERVIEW

This supplementary material documents the prompt template, representative ROS log entries, and synchronized camera frames used by the predictive feedback monitor in a manipulation run. The material is intended to support transparency about the vision-language feedback loop rather than introduce additional quantitative claims.

2 S2. PLANNER-SIDE FEEDBACK INPUT

The planner log records a structured plan with a single plan-level fallback action and a feedback input for the current action:

```
Initial plan: {
  'reason': 'The robot needs to sequentially pick up all cubes ...',
  'plan': ['pick(blue)', 'place()', 'pick(purple)', 'place()',
           'pick(green)', 'place()', ..., 'done()'],
  'fallback_action': 'pitch_retract(0.785398)',
  'feedback_input': {
    'action': 'pick(blue)',
    'future_preconditions': [
      'pick(blue): trajectory_clear may fail if other robot arm
       enters workspace in the future'
    ]
  }
}
```

The same planner log later records cancellation, execution of the fallback action, and replanning:

```
check_for_result returned: TaskResult.CANCELED
Execution of : pick was cancelled
Executing fallback action
New plan: ['pick(purple)', 'place()', 'pick(green)', 'place()',
           'pick(orange)', 'place()', ..., 'done()']
```

3 S3. FEEDBACK PROMPT TEMPLATE

The manipulation feedback prompt asks the VLM to provide an ordinal risk assessment for the current action and its future preconditions:

```
Current action and preconditions: {feedback_input}

You are a robot forecaster. Your goal is to predict if any
precondition for the current action will fail or be at risk
```

Table S1. Representative feedback-node log entries matched to the nearest exported synchronized camera frame. Time offset is image timestamp minus ROS log timestamp.

Event	Log time	Frame	Image time	Offset (s)
`pick(red)` task received	1768216693.911845	000098	1768216693.930319	+0.018
`place()` task received	1768216731.409070	001232	1768216731.405282	-0.004
`improbable` output	1768216742.693707	001574	1768216742.701491	+0.008
`pick(black)` task received	1768216750.433425	001808	1768216750.434794	+0.001

```
within the NEXT SECOND based on predicted agent motion.

Key Focus Areas: Analyze proximity, motion direction, and
workspace contention.

Is there a credible risk of path overlap, occlusion, or
interference within the next second?

IMPORTANT: For each precondition, assess the risk and select
EXACTLY ONE category that best describes the situation.

Categories: very likely, likely, neutral, improbable, very improbable.
```

4 S4. FEEDBACK-NODE OUTPUTS

The feedback log records six task contexts:

```
pick(purple): cube_available may fail if the other robot picks
the purple cube first.
pick(orange): cube_available may fail if the other robot targets
and successfully grasps the orange cube first.
place(): trajectory_clear may fail if the other robot moves its
arm into the path to the target basket.
pick(red): cube_available may fail if the other robot targets
and successfully grasps the red cube first.
place(): trajectory_clear may fail if the other robot moves its
arm into the path to the target basket during placement.
pick(black): cube_available may fail if the other robot targets
and successfully grasps the black cube first.
```

Across the log, 668 parsed ordinal risk outputs were recorded:

```
283 Failure probability: very improbable (VERY_IMPROBABLE)
188 Failure probability: improbable (IMPROBABLE)
196 Failure probability: neutral (NEUTRAL)
  1 Failure probability: very likely (VERY_LIKELY)
```

The synchronized image export begins at timestamp 1768216690.68. Earlier feedback entries, including the beginning of the `pick(purple)` and `pick(orange)` contexts, therefore do not have corresponding exported frames in this dataset. The later entries in Table S1 are matched to frames within milliseconds.

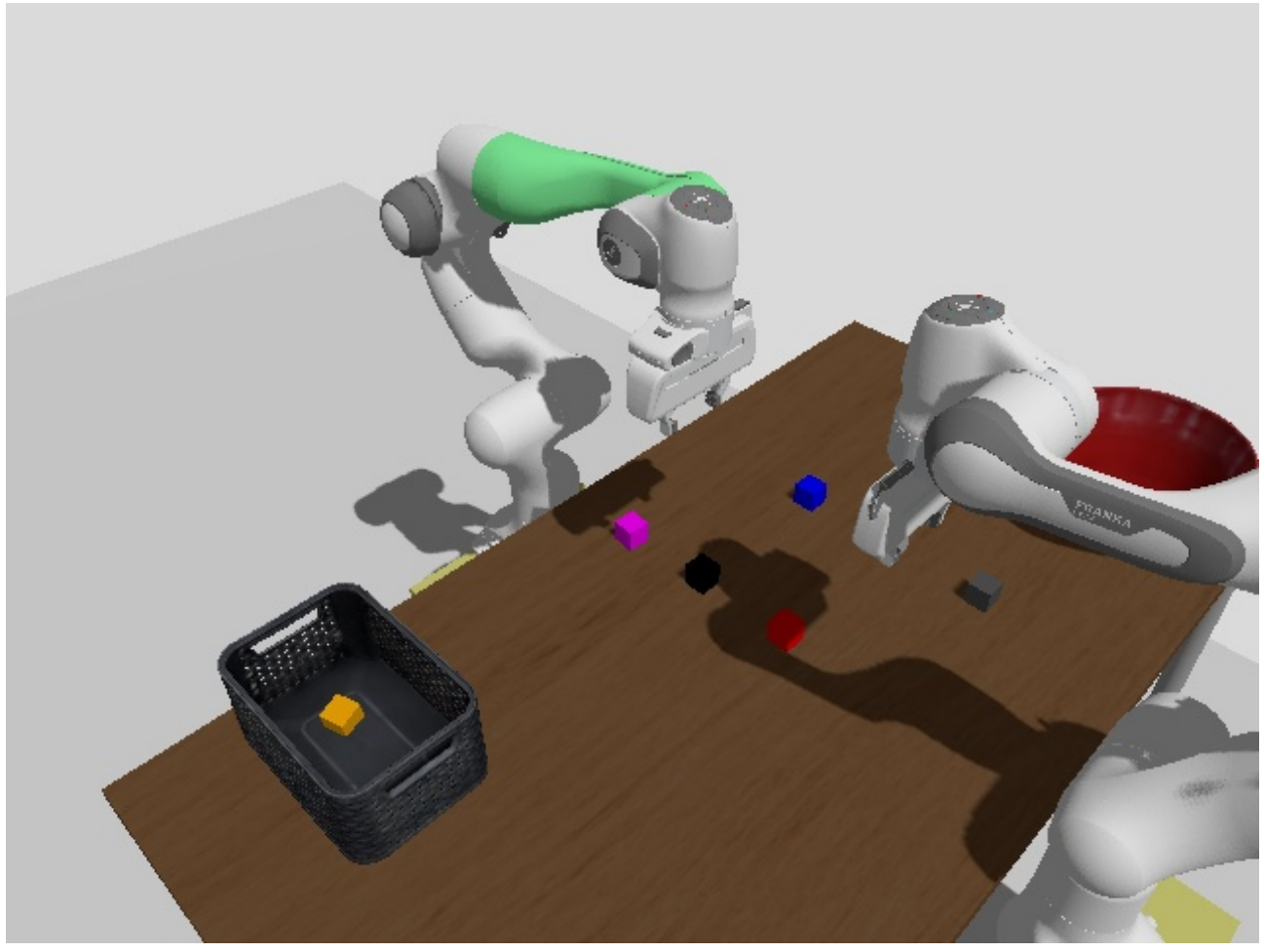

(A) `pick(red)` task context.

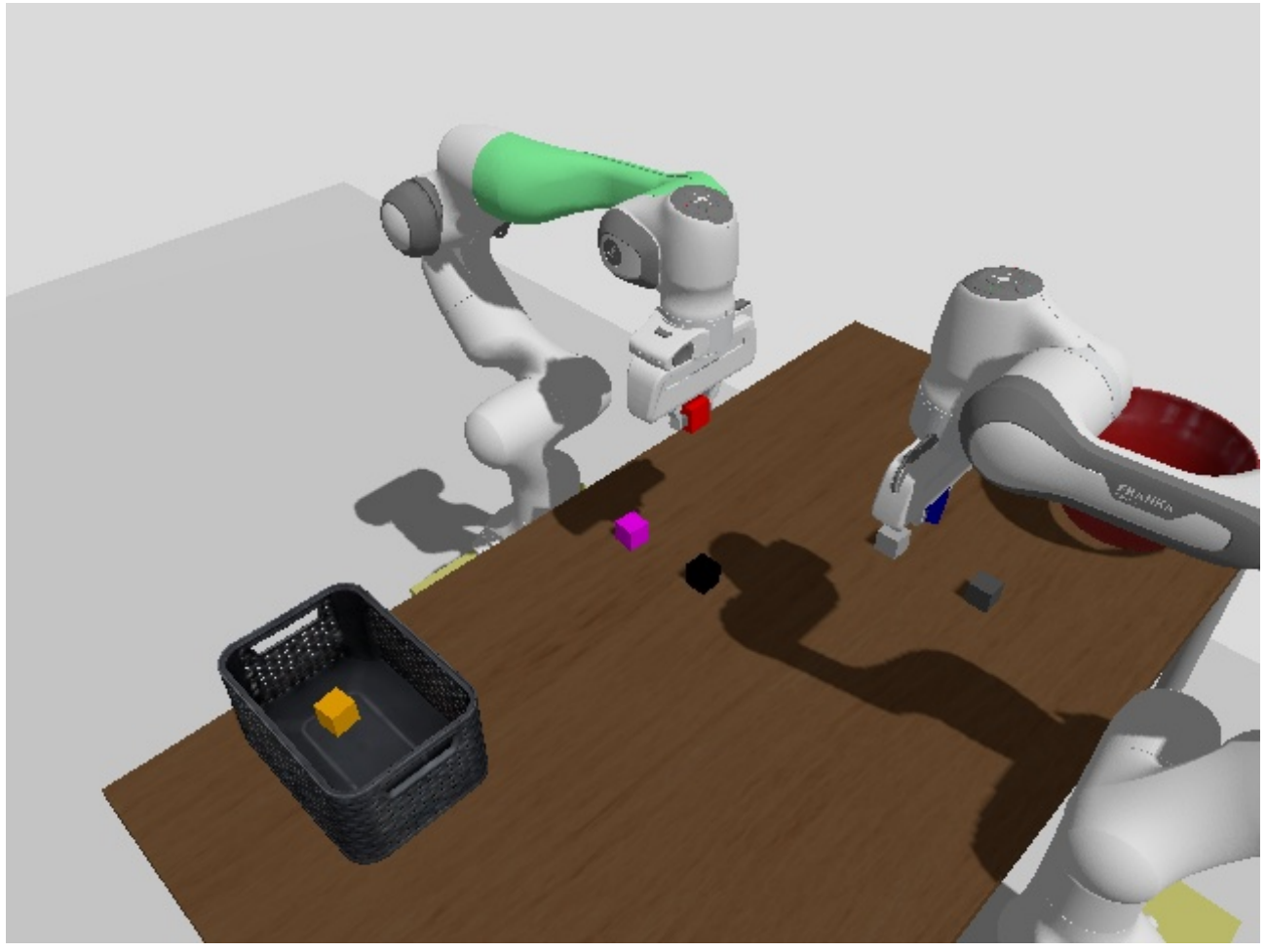

(B) `place()` trajectory-clear context.

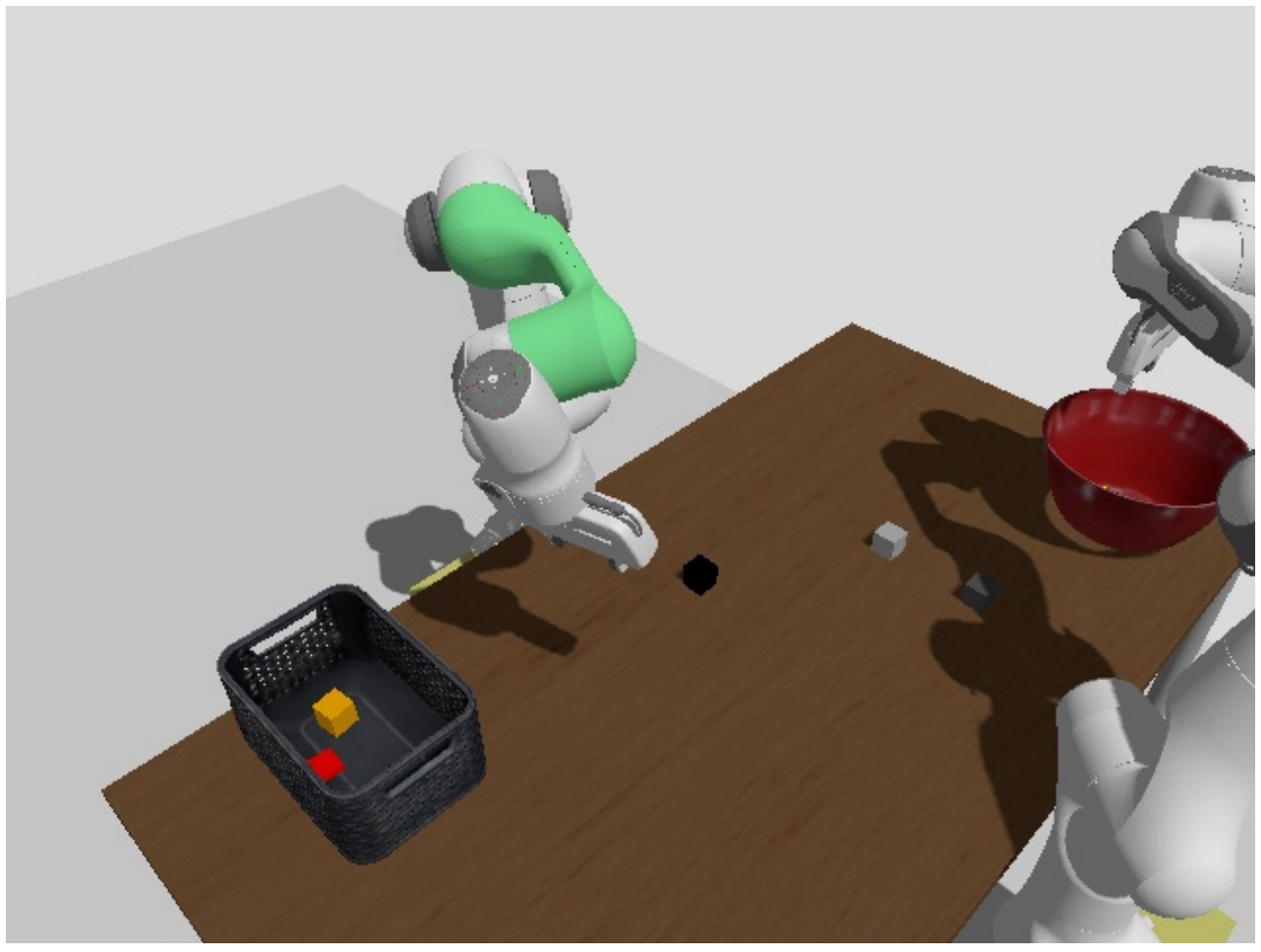

(C) Frame near an `improbable` risk output.

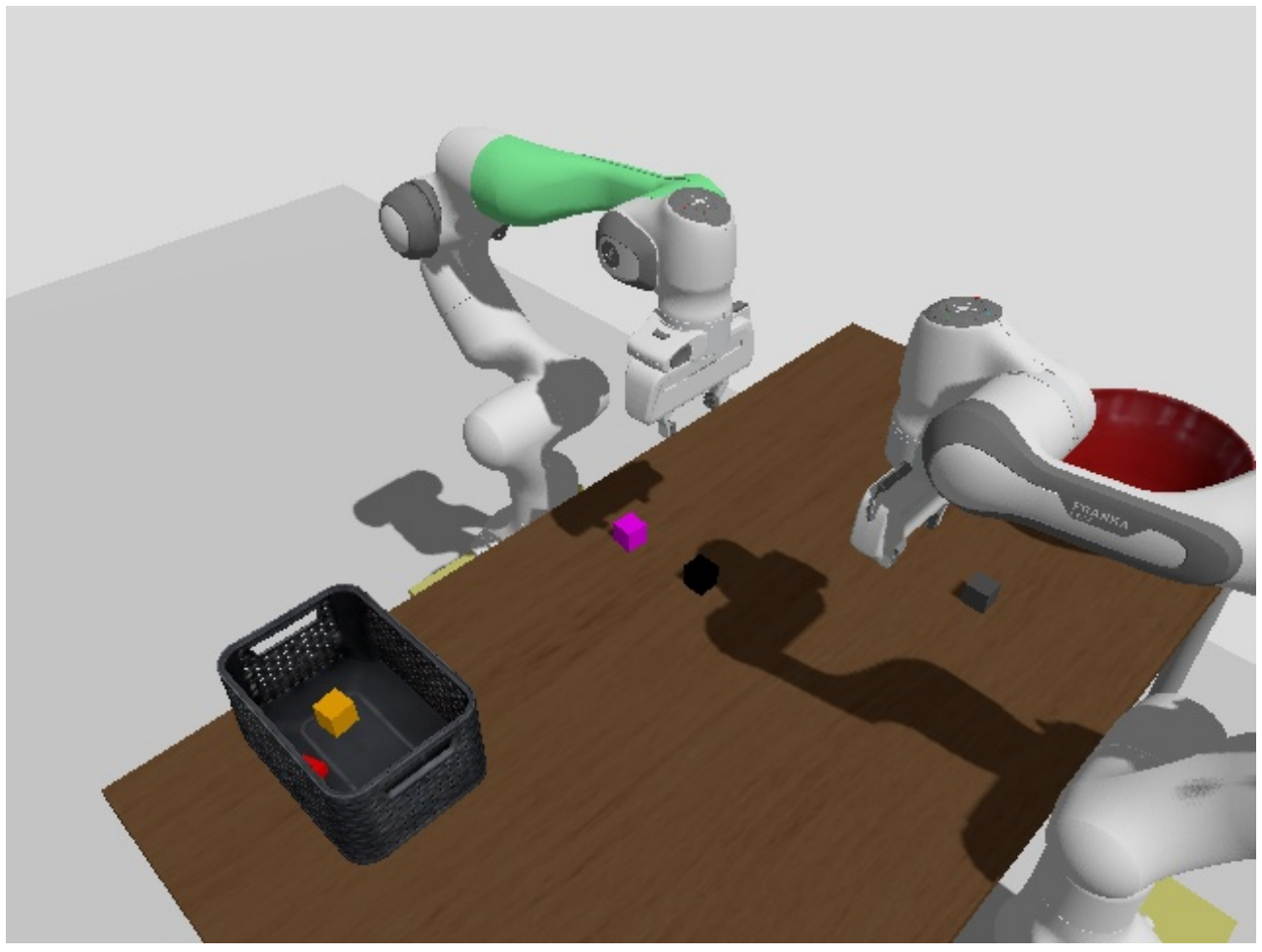

(D) `pick(black)` task context.

Figure S1. Synchronized camera frames matched to representative feedback-node log entries in Table S1.
